# The Enigma We Answer by Living

**DOI:** 10.3201/eid1112.AD1112

**Published:** 2005-12

**Authors:** Alison Hawthorne Deming

**Keywords:** poem, enigma, Einstein

Einstein didn't speak as a child
waiting till a sentence formed and
emerged full-blown from his head.
I do the thing, he later wrote, which
nature drives me to do. Does a fish
know the water in which he swims?
This came up in conversation
with a man I met by chance,
friend of a friend of a friend,
who passed through town carrying
three specimen boxes of insects
he'd collected in the Grand Canyon—
one for mosquitoes, one for honeybees,
one for butterflies and skippers,
each lined up in a row, pinned and labeled,
tiny morphologic differences
revealing how adaptation
happened over time. The deeper down
he hiked, the older the rock
and the younger
the strategy for living in that place.
And in my dining room the universe
found its way into this man
bent on cataloguing each innovation,
though he knows it will all disappear—
the labels, the skippers, the canyon.
We agreed then, the old friends and the new,
that it's wrong to think people are a thing apart
from the whole, as if we'd sprung
from an idea out in space, rather than emerging
from the sequenced larval mess of creation
that binds us with the others,
all playing the endgame of a beautiful planet
that's made us want to name
each thing and try to tell
its story against the vanishing.

**From Genius Loci by Alison Hawthorne Deming**

Copyright 2005 by Alison Hawthorne Deming. Used by permission of Penguin, a division of Penguin Group (USA) Inc.

